# Do the dimensions of the hard palate have a relationship with the volumes of the upper airways and maxillary sinuses? A CBCT study

**DOI:** 10.1186/s12903-021-01724-8

**Published:** 2021-07-20

**Authors:** Murilo Miranda-Viana, Deborah Queiroz Freitas, Alessiana Helena Machado, Amanda Farias Gomes, Yuri Nejaim

**Affiliations:** 1 Department of Oral Diagnosis - Oral Radiology Area, Piracicaba Dental School, University of Campinas, Piracicaba, SP Brazil; 2 Oral Radiology Area, Dental School, Federal University of Mato Grosso Do Sul, Campo Grande, MS Brazil

**Keywords:** Cone-beam computed tomography, Hard palate, Maxillary sinus, Pharynx

## Abstract

**Background:**

As the hard palate is a central structure of the skull, and its close relationship with the nasal cavity, oral cavity, and maxillary sinuses, it would be of interest to study if there is a relationship between this bone and other structures of the stomatognathic system. Thus, this study aimed to assess the dimensions of the hard palate and associate them with sex, and skeletal and breathing patterns. Also, to investigate if there is a relationship between these dimensions and the volumes of the upper airways and maxillary sinuses.

**Methods:**

Two hundred and ninety-eight CBCT scans of patients were classified according to sex, and skeletal and breathing patterns. Then, the linear dimensions of width and height of the hard palate at the regions of the first premolars and first molars, and the volumes of the upper airways and maxillary sinuses were measured using the CS 3D Imaging and ITK-SNAP software, respectively. Data were submitted to multi-way analysis of variance and linear regression, with a significance level of 5% (α = 0.05).

**Results:**

Sex and facial type influenced the hard palate dimensions (*p* < 0.05). Males had greater width and height of the hard palate than females (*p* < 0.0001). It was observed greater width for brachycephalics at the first premolars region (*p* = 0.0032), and greater height for dolichocephalics at the first premolars (*p* = 0.0154) and first molars (*p* = 0.0038) regions. Skeletal malocclusion and breathing pattern did not influence the measurements of the hard palate (*p* > 0.05). There was a significant relationship between the width and height of the hard palate at the premolar’s region and the total volume of the upper airways (*p* = 0.018, and *p* = 0.038), and between both dimensions of the hard palate at the molar’s region and the total volume of the maxillary sinuses (*p* < 0.0001).

**Conclusions:**

The hard palate dimensions are influenced by sex and facial type, but not by skeletal malocclusion or breathing pattern. Also, there is an association between these dimensions and the volumes of the upper airways and maxillary sinuses.

## Background

The hard palate is located at the lower third of the skull, and is composed by the palatal process of the maxilla and the horizontal plate of the palatal bone. This bone plays an important role in the craniofacial complex, as it is involved in orofacial functions such as chewing, swallowing, phonation and breathing [1].

Studies in the literature have reported that the morphology of the hard palate may be influenced by the muscular tension, which can vary with the facial type, and skeletal and breathing pattern of the individual [2–4]. The characteristic stretched and weaker musculature of dolichofacial and mouth-breathing patients, for example, exert less pressure on the bone tissues, leading to possible alterations in the development of the craniofacial complex, including the maxilla and the hard palate [4–6]. Given the central location of the hard palate and its close relationship with the nasal cavity, oral cavity, and maxillary sinuses, it is possible that changes in its morphology may also affect other structures of the stomatognathic system [7–9].

Only few researches [6–8] had the hard palate as an object of study, and most of them were performed with small samples and in pediatric patients—a period in which the bones and integumentary tissues are not yet fully developed—and/or in syndromic patients, restricting the obtained conclusions to these specific groups. Additionally, all these studies were conducted in bi-dimensional radiographs, which present overlapping images and magnification/distortion of structures, or in plaster models, which may present distortions inherent to the molding process or to the plaster itself. Therefore, studies on the hard palate, using modalities of exams with higher accuracy in the evaluation of the craniofacial structures, such as cone beam computed tomography (CBCT), are of interest [10]. To the best of the authors knowledge, there are no studies on the evaluation of a possible relationship between the hard palate and the volumes of the maxillary sinuses and/or upper airways.

Therefore, the aim in this study was to assess the dimensions the hard palate and associate them with sex, and skeletal and breathing patterns. Also, to investigate if there is a relationship between these dimensions and the volumes of the upper airways and maxillary sinuses.

## Methods

This observational, cross-sectional, and retrospective research was initiated after approval by the local institutional review board (IRB) under the protocol number: **3.491.476**.

### Sample selection

Initially, 340 cone beam computed tomography (CBCT) scans with extended FOV (field of view) and pertaining to the database of oral radiology clinic of Piracicaba Dental School (São Paulo, Brazil). The images were obtained prior to the present research (January 2014 to December 2016), and for clinical reasons not related to it. Images of patients 18 years old or older, of both sexes, with all teeth present (third molars were not required), were included in the sample. CBCT scans of patients with history of trauma or orthognathic surgery, presence of bone fracture, syndromes, bone exostoses, pathological lesions, or cleft lip/palate, as well as scans with presence of artifacts impairing the evaluation of the anatomical structures of the head and neck, were excluded from the sample.

Hence, after employing the exclusion and inclusion criteria, the final sample for the morphometric assessment of the hard palate, and the volumetric analysis of the upper airways, was composed of 298 CBCT scans—144 males (18 to 64 years old, mean age 32.04 ± 12.48 years), and 154 females (18 to 76 years old, mean age 30.87 ± 11.47 years). Due to limitations that hampered the volumetric evaluation of the maxillary sinuses (e.g., mucous disease), only 212 of the 298 CBCT scans were used for this analysis—104 males (18 to 64 years old, mean age 32.40 ± 12.46 years), and 108 females (18 to 76 years old, mean age 29.62 ± 11.44 years).

All CBCT images were acquired with an i-CAT® Next Generation device (Imaging Sciences International, Hatfield, Pa), with the following acquisition parameters: 5 mA, 120 kVp, 17.3 s scanning time, 0.3 mm^3^ voxel size, and an extended field of view (FOV)—23 × 17 cm. The images were exported in digital imaging and communications in medicine files (DICOM) format and selected in XoranCat® Software version 3.1.62.

### Sample classification

Initially, each patient was classified in regard to skeletal patterns—skeletal malocclusion (Class I, II, or III), and facial type (dolichocephalic, brachycephalic, or mesocephalic), and breathing pattern (nasal breathing, or mouth breathing). To do so, two previously calibrated examiners assessed in consensus the multiplanar reconstructions obtained from the CBCT images, using the Carestream Dental 3D Imaging software (version 3.10.9.0, Atlanta, Georgia, USA).

**Fig. 1:**
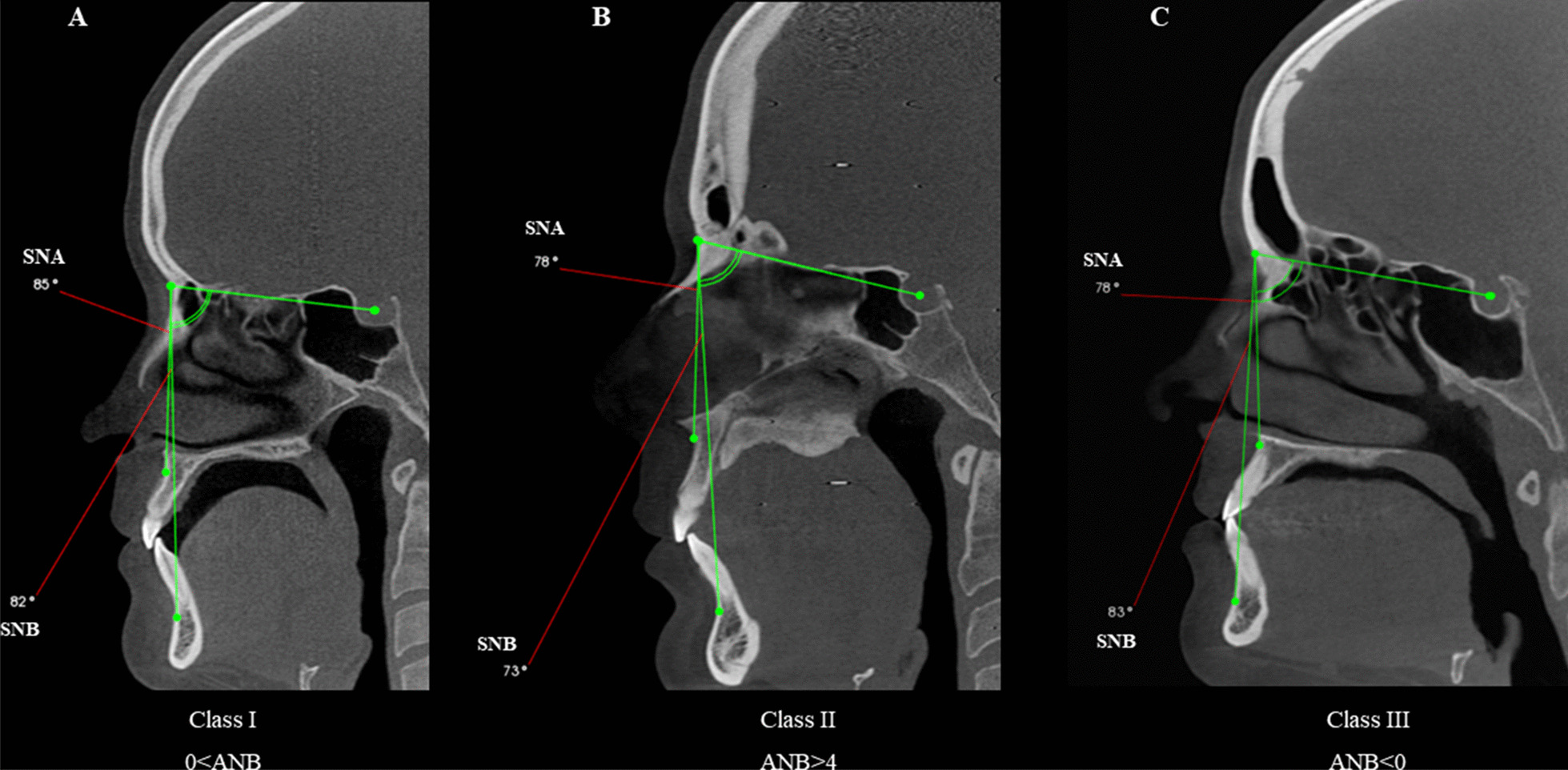
Sagittal reconstructions of cone-beam computed tomography demonstrating patients’ classification according to skeletal malocclusion, based on Steiner’s cephalometric standards for the SNA, SNB, and ANB angles. *SNB-SNA* ANB. **A** Class I: 0 < ANB < 4, **B** Class II: ANB > 4, **C** Class III: ANB < 0

**Fig. 2:**
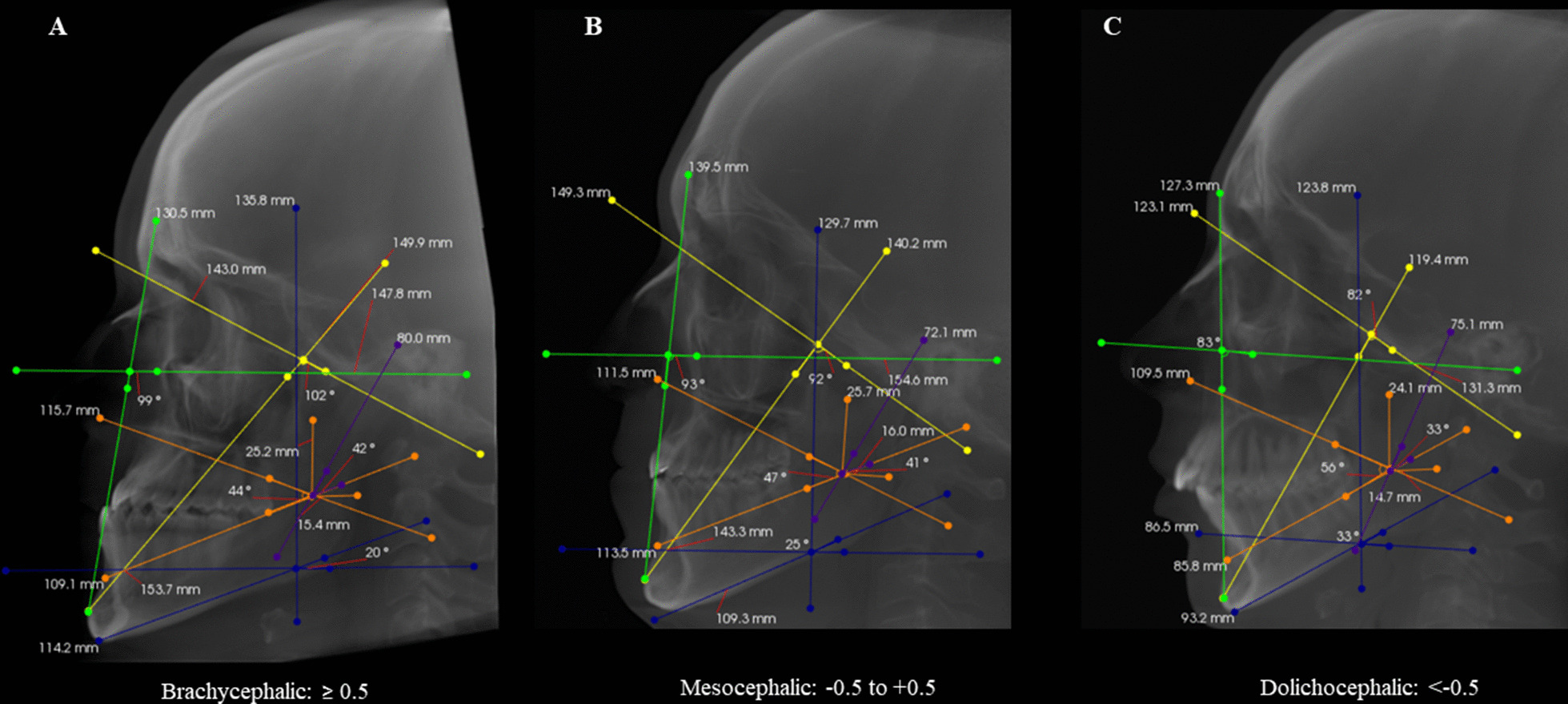
Sagittal reconstructions of cone-beam computed tomography demonstrating patients’ classification according to facial type, based on Vert index. Representative colors of cephalometric measurements: yellow: facial axis; green: facial depth; blue: mandibular plane; orange: lower facial height; and purple: mandibular arch. **A** Brachycephalic: ≥ 0.5, **B** Mesocephalic: − 0.5 to + 0.5, **C** Dolichocephalic: < −  0.5

Skeletal malocclusion was established based on Steiner’s cephalometric standards for the SNA, SNB, and ANB angles. The ANB angle was obtained by subtracting the SNB from the SNA values (ANB = SNA – SNB). ANB values 0 to 4 = skeletal Class I; ANB > 4 = Class II; and ANB < 0 = Class III (Fig. 1). [11, 12] Facial type was determined based on the Vert index, [13] which was obtained from the arithmetic average of five cephalometric measures: facial depth (Po-Or/N-Pog), facial axis angle (N-Ba/Pt-Gn), lower facial height (Xi-ANS/Xi-Pm), mandibular plane angle (Go-Me/Po-Or), and mandibular arch (Dc-Xi/Xi-Pm). Resultant values greater than 0.5 determined the brachycephalic type; values lower than − 0.5 determined the dolichocephalic type; and values between − 0.5 and + 0.5 represented the mesocephalic type (Fig. 2). Table 1 shows the definitions of the variables.

**Table 1:** Definitions of variables in the classification of individuals regarding skeletal patterns (skeletal malocclusions and facial types)

Skeletal pattern	Variables	Definition
Skeletal malocclusion	SNA	Angle formed from the points Sella (S), Nasion (N) and A
	SNB	Angle formed from the points Sella (S), Nasion (N) and B
	ANB	Angle formed from the points A, Nasion (N) and B
Facial type	Facial depth	Angle formed by intersecting the lines Po (Porion)—Or (Orbitale) and N (Nasion)—Pog (Pogonion)
	Facial axis angle	Angle formed by intersecting the lines N (Nasion)—Ba (Basion) and Pt (Pterygoid Point)—Gn (Gnathion)
	Lower facial height	Angle formed by intersecting the lines Xi (Geometric center of the ramus)—ANS (Anterior nasal spine) and Xi (Geometric center of the ramus)—Pm (Suprapogonion)
	Mandibular plane angle	Angle formed by intersecting the lines Go (Gonion)—Me (Menton) and Po (Porion)—Or (Orbitale)
	Mandibular arch	Angle formed by intersecting the lines Dc (Point in the center of the mandibular condyle)—Xi (Geometric center of the ramus) and Xi (Geometric center of the ramus)—Pm (Suprapogonion)

**Fig. 3:**
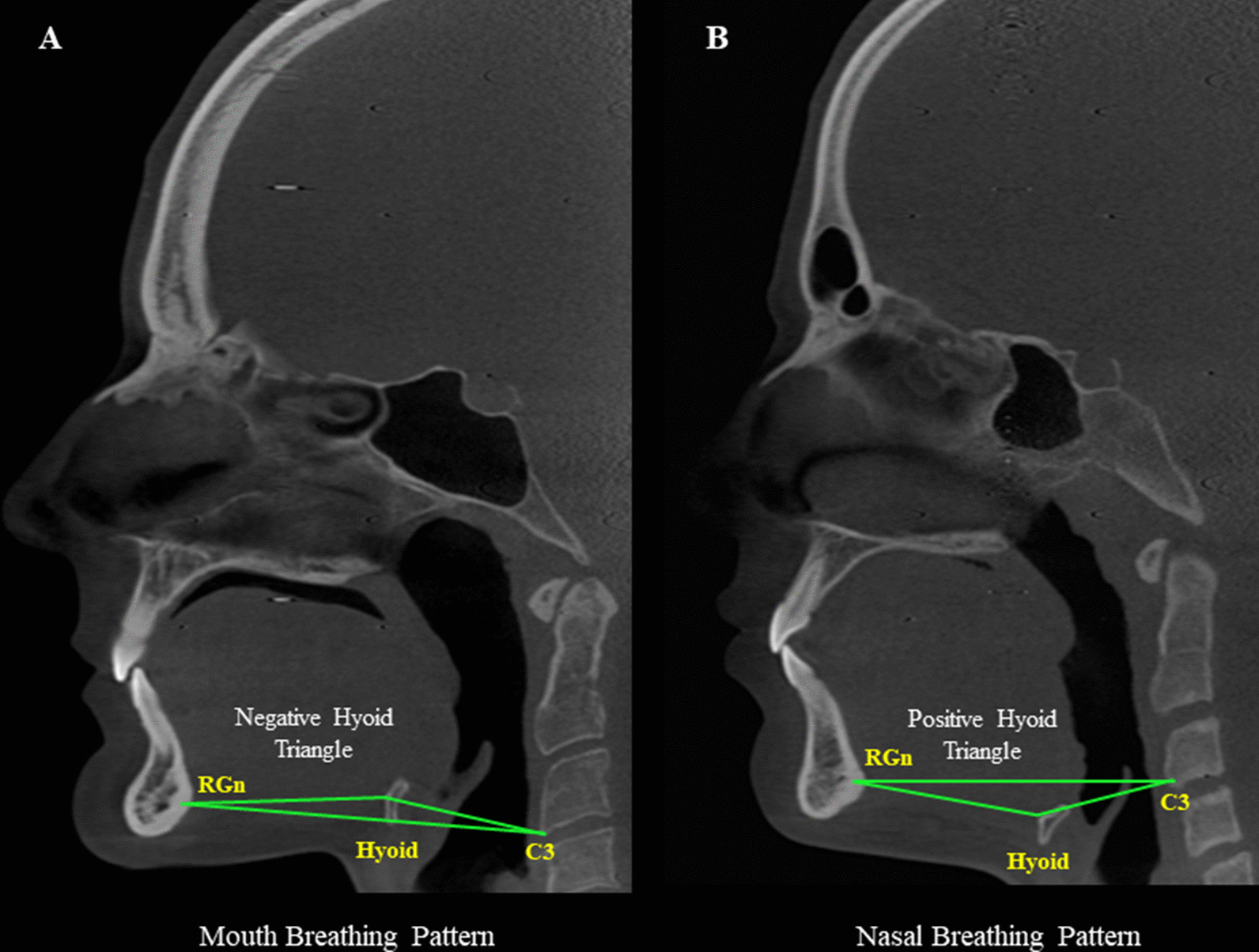
Classification of patients by breathing pattern based on the hyoid triangle. **A** Mouth Breathing Pattern, **B** Nasal Breathing Pattern

Regarding the breathing pattern, the classification was based on the hyoid triangle methodology, which considers the position of the hyoid bone (Fig. 3). [14–16] Firstly, each CBCT scan was spatially re-oriented so the software’s vertical reference line was placed in the median sagittal plane, in the coronal view; then, the horizontal reference line in the sagittal reconstruction, and the vertical reference line in the axial reconstruction were positioned passing through the anterior and posterior nasal spines for standardization [14]. Thus, in the sagittal view, the hyoid bone was clearly visualized. A line between the most inferior-anterior point of the third cervical vertebra (C_3_) and the most posterior point of the mandibular symphysis (retrognathic cephalometric point—RGn) was drawn, establishing the triangle basis. Another line from C_3_ to the most anterior point of the hyoid bone was drawn, and then to the RGn point, determining the hyoid triangle. Thus, if the hyoid bone was positioned on or above the RGn-C_3_ plane, that meant a higher position of the hyoid, determining a negative triangular position and, therefore, a mouth breathing pattern. Conversely, if the hyoid bone was positioned bellow the RGn-C_3_ plane, that meant a lower position of the hyoid, determining a positive triangular position and, therefore, a nasal breathing pattern.

**Fig. 4:**
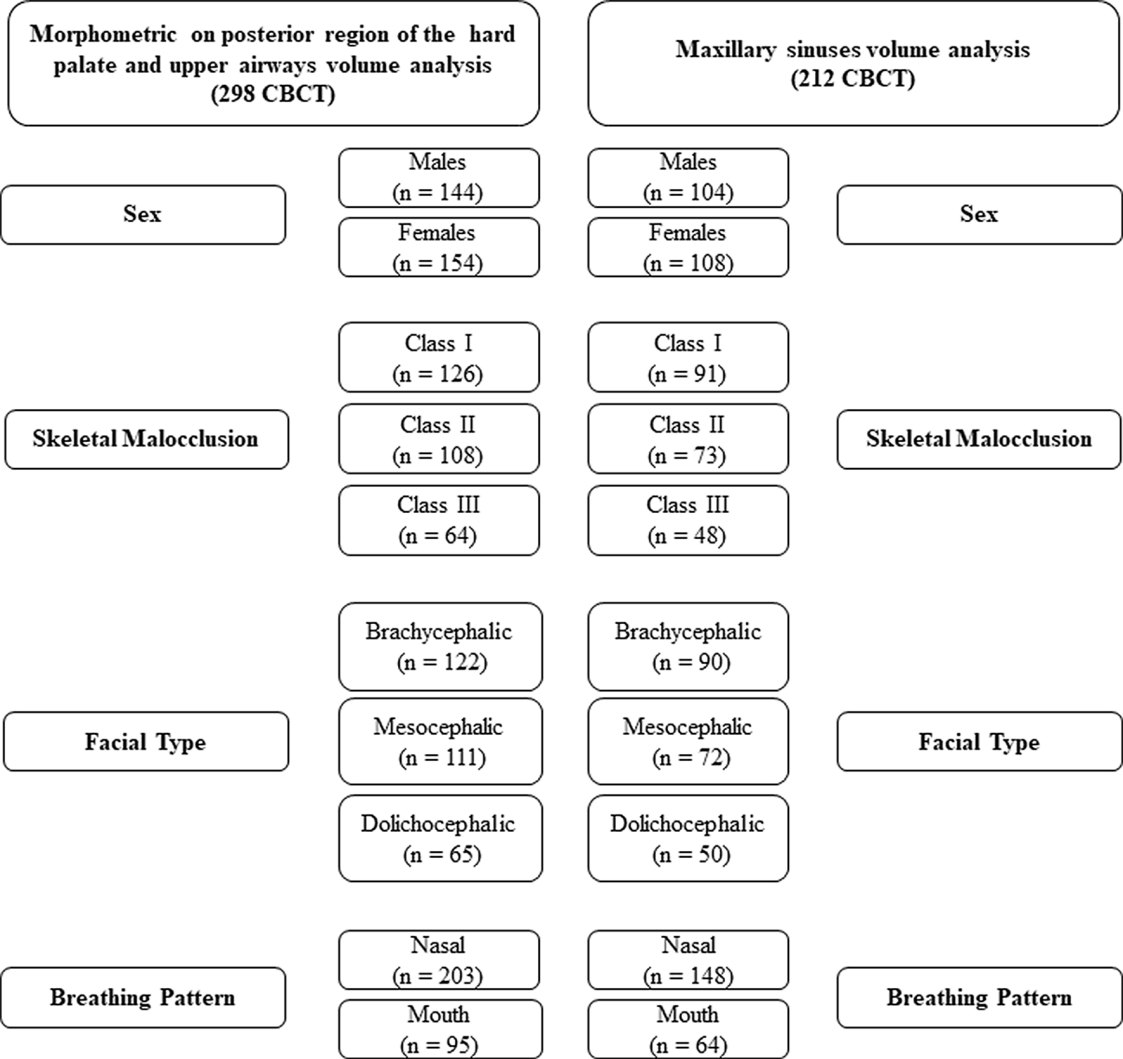
Diagram of the sample after all classifications have been established

Sample distribution according to the classifications is shown in Fig. 4.

### Three-dimensional assessment

The Carestream Dental 3D Imaging software (version 3.10.9.0, Atlanta, Georgia, USA) was used for the morphometric analysis of the hard palate, and the ITK-SNAP v.3.0 (Cognitica, Philadelphia, PA) was employed to evaluate the volumes of the upper airways and maxillary sinuses.

Before initiating the evaluations, the examiners were instructed about the assessment of the hard palate, upper airways, and maxillary sinuses, using scans that were not included in the final sample as examples. Two examiners (3 years of experience in the assessment of CBCT images) evaluated the scans independently. The evaluations were performed in a low-light and silent environment, using a 24.1-in LCD display (Barco MDRC-2124; Barco., Kortrijk, Belgium) with a resolution of 1920 × 1200 pixels.

#### Morphometric analysis of the hard palate

**Fig. 5:**
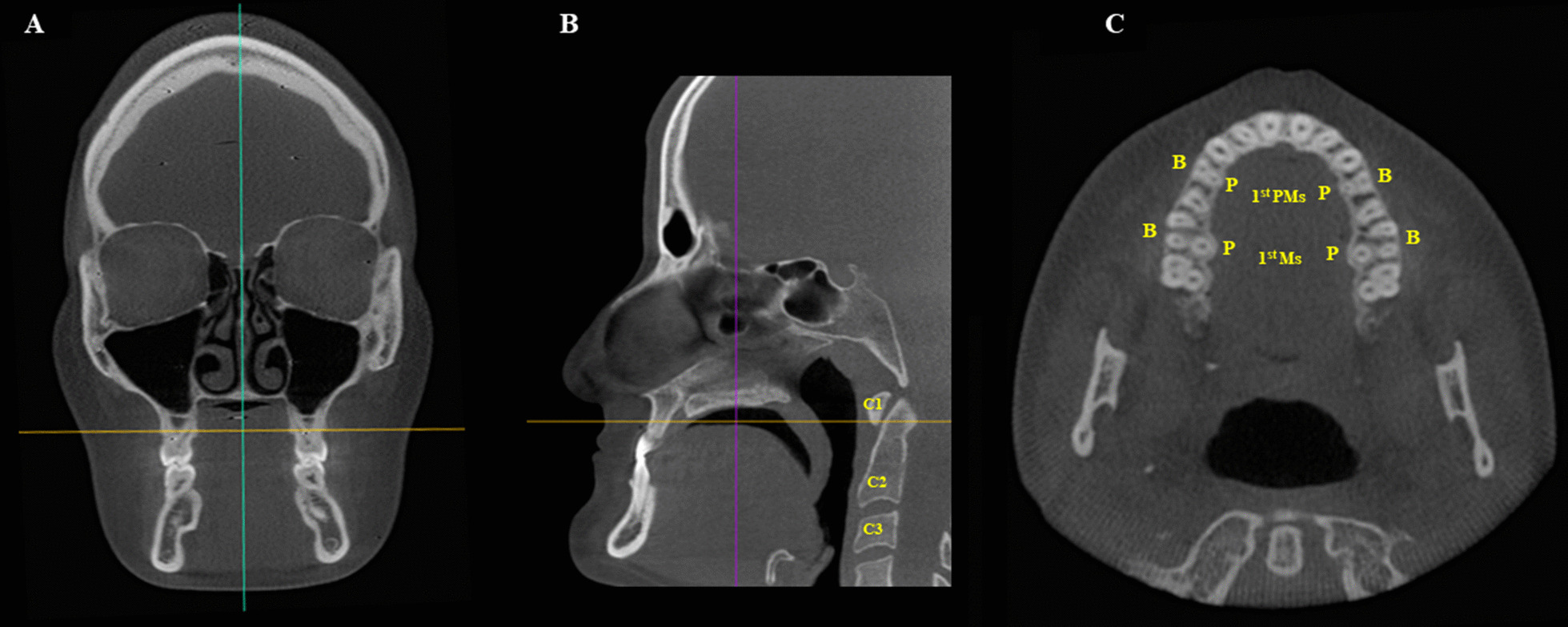
Coronal section orientation in the morphometric evaluation of the hard palate. **A** Coronal reconstruction—green line positioned at the median sagittal plane and perpendicular to the yellow line, which is placed over the teeth of the maxilla, **B** Sagittal reconstruction—Yellow line passing through the lowest point of the first cervical vertebra (C_1_); **C** Axial reconstruction—maxillary arch showing the individualized roots of the multiradicular teeth. C_2_: second cervical vertebra; C_3_: third cervical vertebra; B: buccal; P: palatine; 1st PMs: first premolars; 1st Ms: first molars

Initially, each CBCT scans was spatially reoriented to standardize the assessment. As the literature does not present a study with a methodology for evaluating the upper teeth roots, the reorientation was arbitrarily developed by the authors. The vertical reference line of the software was placed at the median sagittal plane, in the coronal view; then, in the sagittal reconstruction, the horizontal reference line was positioned passing through the lowest point of the first cervical vertebra, with the roots of all teeth being observed in the axial view. Measurements on the posterior region of the hard palate were done at the levels of the upper first premolars, and first molars (Fig. 5).

**Fig. 6:**
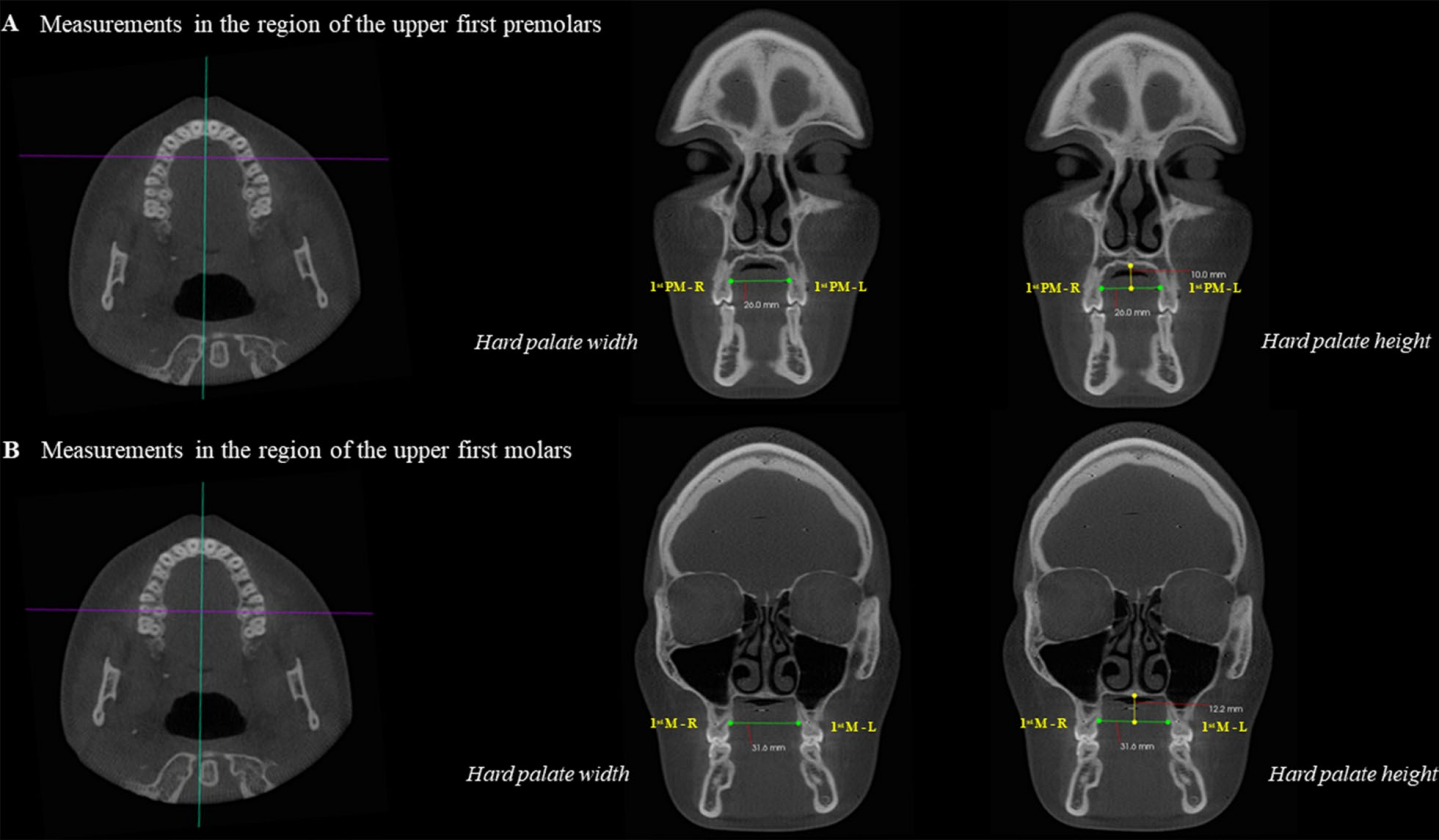
Morphometric analysis of the hard palate. **A** and **B** Axial reconstructions—green line positioned in the median sagittal plane and perpendicular to the purple line, which passes over the palatine roots of the upper first premolars and first molars. **A** Coronal reconstruction—measurement of hard palate width (green line) and height (yellow line) at the first premolars region. **B** Coronal reconstruction—measurement of the hard palate width (green line) and height (yellow line) at the first molars region. 1^st^ PM first premolar; 1st Ms: first molar; R: right; L: left.

To perform the measurements of the hard palate at the level of the first premolars, in the axial view, the horizontal reference line of the software was positioned on the palatal roots of the first premolars, crossing the right and left sides of the palate. Then, in the coronal view, with the measuring tool of the software, a horizontal line connecting the palatal bone cortex at the region of the right first premolar to the palatal bone cortex at the region of the left first premolar was drawn, determining the values of posterior width of the hard palate at this level. The same procedure was performed at the first molars level. Furthermore, the height of the hard palate at the levels of the first premolars and first molars was also measured. To do so, a line perpendicular to the horizontal line previously described (posterior width) was established from the most superior point of the hard palate to the center of the horizontal line (Fig. 6).

#### Volumetric analysis

The volumetric analysis of the upper airways and maxillary sinuses were performed by semiautomatic segmentation.

**Fig. 7:**
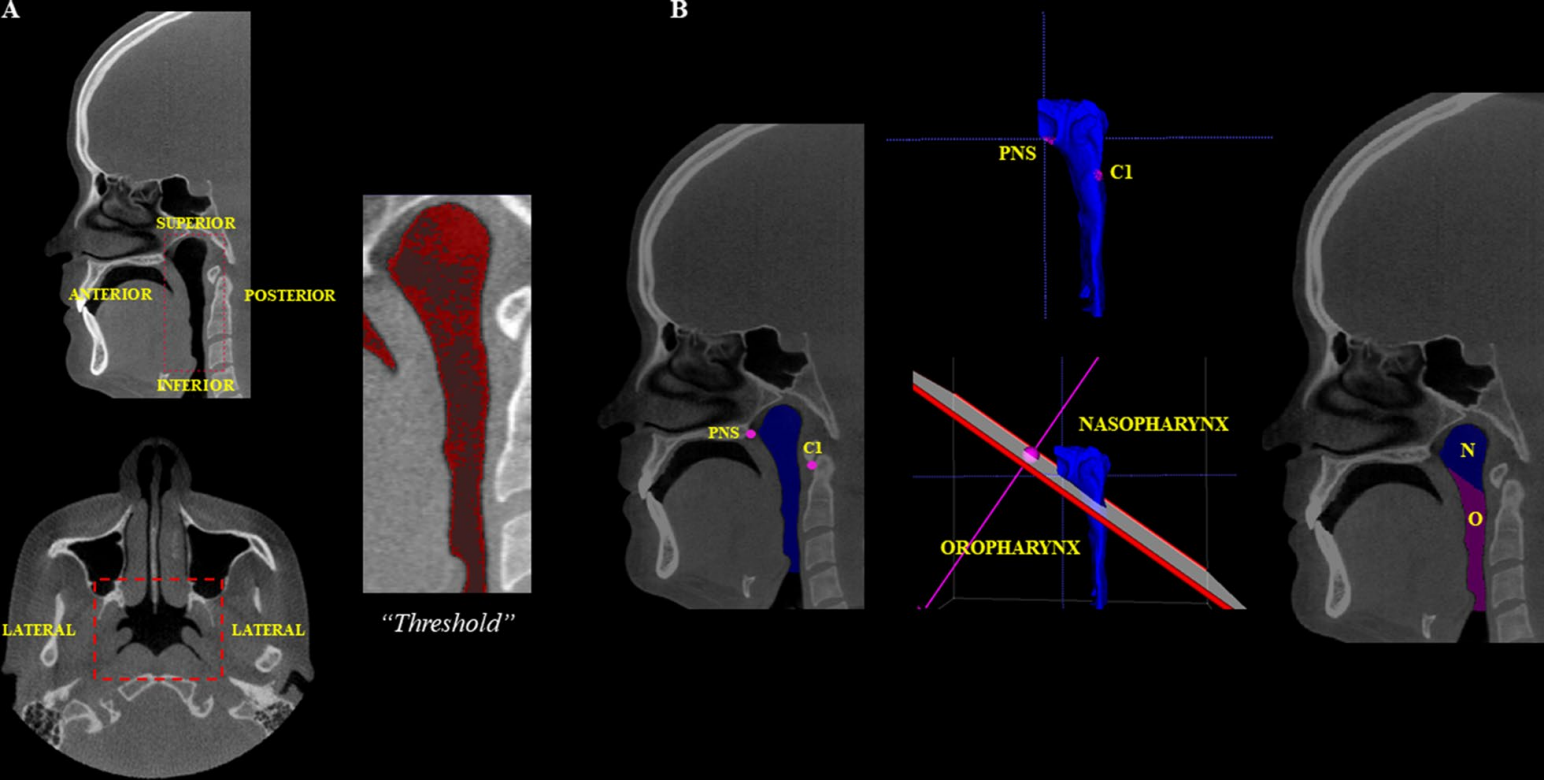
Volumetric analysis of the upper airways. **A** Sagittal and axial reconstructions—Region of Interest (ROI) for upper airway segmentation, and threshold selection for semi-automatic airway filling. **B** Sagittal reconstructions—Upper airways filled, and segmentation for individualization of the nasopharynx (N)—blue region, and oropharynx (O)—pink region. PNS: posterior nasal spine; C1: first cervical vertebra

In the evaluation of the upper airways, the volumes of the nasopharynx, oropharynx, and of the entire upper airway were analyzed. Firstly, the region of interest (ROI) for segmentation was established according to the following limits: the anterior limit running along the posterior nasal spine, parallel to the sagittal plane; posterior limit passing through the cervical vertebra (C_2_ and C_3_); lateral limits running along the lateral walls of the pharynx; inferior limit passing tangentially to the medial caudal projection of the third cervical vertebra (C_3_), perpendicular to the sagittal plane; and, the superior limit up to the highest point of the nasopharynx [17]. After establishing the ROI, the segmentation was performed by means of three interactive steps: firstly, a threshold range was set to determine the start and end of the segmentation process. A value of − 1000 was set for the lower threshold, and a value varying from − 660 to − 531 was established for the upper threshold, meaning that all voxels with gray values inside that interval were selected to construct the 3D model. After that, “seeds” were placed in the ROI to initiate the segmentation; and lastly, the segmentation evolution was run by selecting its velocity and end. If there was an area that was not defined, a manual adjustment was made by the evaluator. To obtain the volumes of the nasopharynx and oropharynx separately, the software’s scalpel tool was used. An oblique cut line was traced over the reference structures: the lower aspect of the first cervical vertebra (C_1_), and the posterior nasal spine. The upper airway’s total volume, and the volumes of the naso and oropharynx regions were calculated in cubic millimeters (mm^3^) by the software (Fig. 7).

**Fig. 8:**
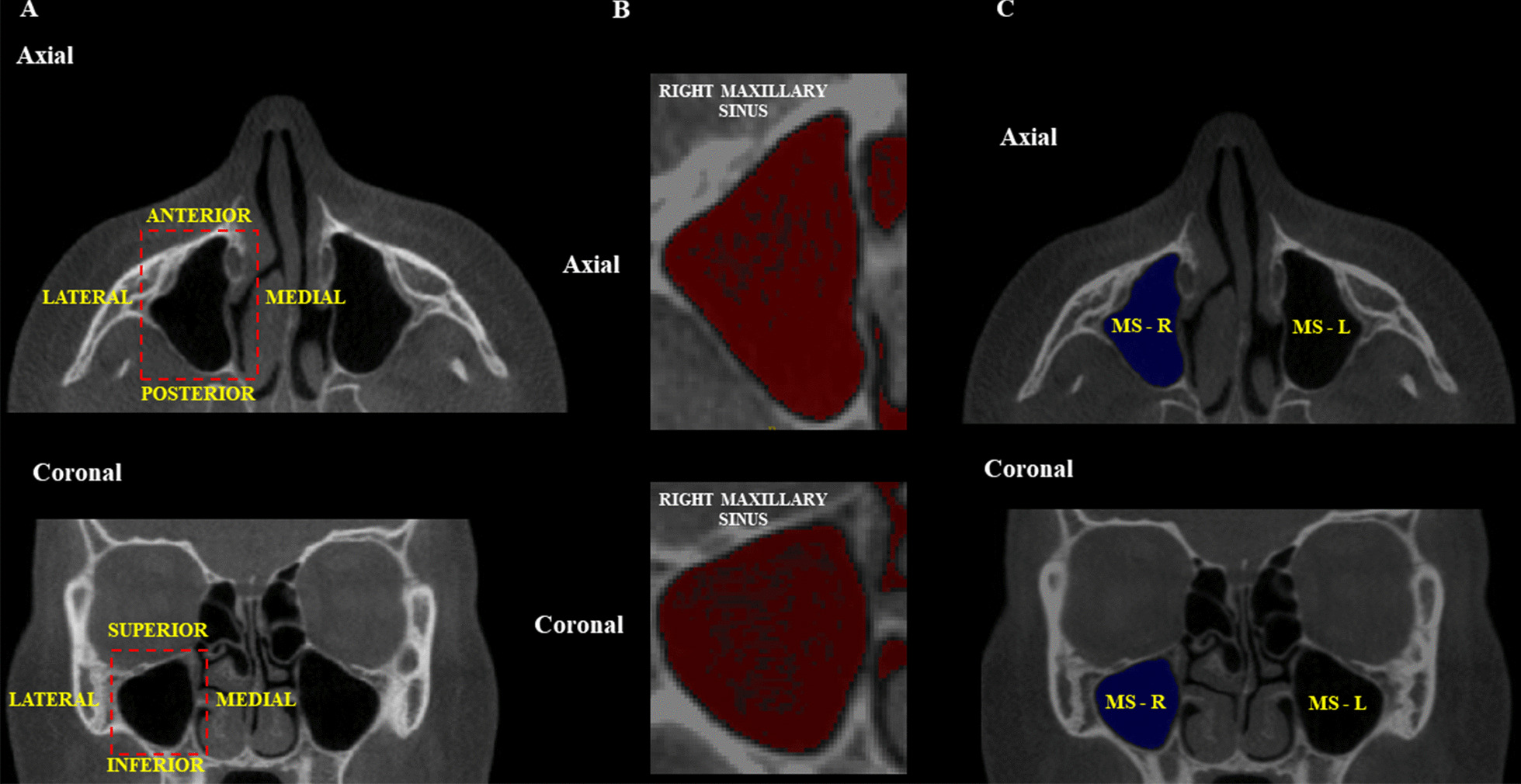
Volumetric analysis of the maxillary sinuses. **A** ROI for segmentation of the maxillary sinuses. **B** Threshold selection for semi-automatic filling of the maxillary sinuses. **C** Filled maxillary sinus to obtain the volume ​​in mm^3^. *MS:* maxillary sinus; *R:* right; *L:* left

Regarding the maxillary sinuses, the volumes of the left and right sinuses were evaluated, and their total volume was obtained. The examiner established the region of interest (ROI) in the CBCT multiplanar reconstructions, using the lateral, medial, superior, inferior, anterior, and posterior walls of the maxillary sinus as limits. [18] Then, the threshold range (lower threshold: − 1000; upper threshold: − 678 to − 518 for the right sinus, and − 710 to − 572 for left sinus) was set, and semiautomatic filling of the selected region was performed after placing the “seeds” along the ROI. A manual adjustment was made if there was an area that was not defined. The total volume of the maxillary sinuses was established in mm^3^ (Fig. 8).

Thirty days after the end of evaluations, part of the sample (30%) was reassessed to obtain the intraexaminer agreement.

#### Data analysis

Intra- and interexaminer agreements were calculated by the intra-class correlation coefficient (ICC) test (> 0.75—excellent; 0.40–0.75—moderate, and < 0.40—poor). [19].

The normality of the data was verified using the Kolmogorov–Smirnov test. The influence of the studied factors (sex, skeletal malocclusion, facial type, and breathing pattern) on the linear measurements of the hard palate in the regions of the first premolars and first molars was tested by the multi-way analysis of variance (ANOVA).

In order to observe the relationship between the linear measurements of the hard palate and the volumes of the upper airways and maxillary sinuses, multiple linear regression was used. To do so, each factor (width and height of the hard palate at the levels of the first premolars and first molars) was subjected to a simple linear regression to verify the possible influence of each of them on the studied volumes. Then, factors showing *p*-values lower than 0.20 (*p* < 0.20) were applied together in a multiple linear regression.

The null hypothesis considered that the dimensions of the hard palate are not influenced by sex, skeletal and breathing patterns. It also considered that there is no relationship between the dimensions of the hard palate and the volumes of the upper airways and maxillary sinuses. All analyses were conducted with the Statistical Package for the Social Sciences v.23.0 (*SPSS* Inc., Chicago, IL, EUA), and the Bioestat® v.5.3 (Instituto de Desenvolvimento Sustentável Mamirauá, Tefé, AM, Brazil) software, with a significance level established at 5% (*p* < 0.05).

## Results

ICC results revealed excellent intra- and interexaminer agreements for all assessments. For the maxillary sinuses volume, the values of intra- and interexaminer agreements ranged from 0.97 to 0.99, and from 0.96 to 0.99, respectively. For the upper airways volume, the intra- and interexaminer agreements ranged from 0.96 to 0.98, and from 0.77 to 0.94, respectively; and for the linear measurements of the hard palate, they ranged from 0.89 to 0.93, and from 0.78 to 0.86.

Table 2 shows the linear measurements of the hard palate according to sex, skeletal patterns, and breathing patterns. There was an influence of sex on the hard palate measurements (*p* < 0.0001), except for the height in the first premolars region (*p* = 0.4563). Males presented greater width of the hard palate in both first premolars and first molars regions, and greater values of height of the hard palate in the first molars region. Regarding the facial types, brachycephalic patients had greater values of hard palate width in the first premolars region (*p* = 0.0032) than the other facial types, while dolichocephalic individuals had greater values of hard palate height than brachycephalics (*p* = 0.0154). For the height of the hard palate in the first molars region, dolichocephalic patients had greater values than the other facial types (*p* = 0.0032). Skeletal malocclusion, and breathing pattern did not influence the measurements of the hard palate (*p* > 0.05).

**Table 2:** Relationship between the linear measurements of the hard palate and sex, and skeletal and breathing patterns

	Linear measurements of the hard palate											
	First premolars region						First molars region					
	Hard palate width			Hard palate height			Hard palate width			Hard palate height		
	Mean (mm)	SD	*p*-value	Mean (mm)	SD	*p*-value	Mean (mm)	SD	*p*-value	Mean (mm)	SD	*p*-value
Sex												
Male	29.018	2.799	*p* < 0.0001*	9.597	3.188	0.4563	36.283	3.440	*p* < 0.0001*	13.663	2.719	*p* < 0.0001*
Female	27.509	2.606		9.100	2.266		33.692	2.904		11.761	2.137	
Skeletal malocclusion												
Class I	28.298	2.647	0.6957	9.153	2.835	0.1646	35.038	3.208	0.6733	12.454	2.563	0.4598
Class II	28.005	2.929		9.827	2.542		34.432	3.528		12.596	2.729	
Class III	28.514	2.882		8.889	2.868		35.621	3.575		13.268	2.450	
Facial type												
Brachycephalic	28.970^A^	2.761	0.0032*	8.782^B^	2.641	0.0154*	35.759	3.349	0.0507	12.200^B^	2.586	0.0038*
Mesocephalic	27.919^B^	2.810		9.438^AB^	2.777		34.677	3.393		12.626^B^	2.388	
Dolichocephalic	27.409^B^	2.550		10.223^A^	2.724		33.869	3.294		13.675^A^	2.783	
Breathing pattern												
Nasal	28.510	2.897	0.4446	9.432	2.761	0.9774	35.315	3.566	0.5095	12.895	2.692	0.5390
Mouth	27.657	2.497		9.146	2.753		34.150	2.964		12.222	2.378	

*SD* Standard deviation

^*^Statistically significant (*p* < 0.05)—ANOVA

Different letters indicate significant difference among facial type for oropharynx volume, according to Post-Hoc de Tukey—ANOVA

Furthermore, in order to observe the relationship between the measurements of the posterior region of the hard palate (width and height) and the volumes of the upper airways and maxillary sinuses, a multiple linear regression analysis was performed (Table 3). It was found an association between the measurements of the hard palate at the first premolars region and the upper airways volume: width and height of the hard palate and the total volume of the upper airway (*p* = 0.018 and 0.038, respectively); width and height of the hard palate and the nasopharynx volume (*p* = 0.013 and 0.035, respectively); and width of the hard palate and the oropharynx volume (*p* = 0.024). In this association, the greater the height of the hard palate, the lower the volume of the upper airways, and the greater the width of the hard palate, the higher the volume of the upper airways. It was also observed an association between the width and height of the hard palate at the first molars region and the total volume of the maxillary sinuses (*p* < 0.0001), in which the greater the width and height of the hard palate, the greater the volume of the maxillary sinuses.

**Table 3:** Multiple linear regression for the association between the volumes of the upper airways and maxillary sinuses and the linear measurements of the hard palate

Multiple linear regression	Factors	BC	SE	CI (95% for B)	*p*-value
*Upper airways*					
Total volume	HP width (1st PM)	0.189	0.197	(0.082–0.857)	0.018*
	HP height (1st PM)	− 0.121	0.147	(− 0.595–− 0.017)	0.038*
	HP width (1st M)	− 0.042	0.159	(− 0.398–0.228)	0.593
Nasopharynx volume	HP width (1st PM)	0.198	0.083	(0.045–0.373)	0.013*
	HP height (1st PM)	− 0.123	0.062	(− 0.255–− 0.009)	0.035*
	HP width (1st M)	− 0.033	0.068	(− 0.162–0.104)	0.669
Oropharynx volume	HP width (1st PM)	0.132	0.105	(0.032–0.446)	0.024*
	HP height (1st PM)	− 0.096	0.107	(− 0.386–0.034)	0.100
*Maxillary sinuses*					
Total volume	HP width (1st M)	0.291	0.173	(0.486–1.169)	*p* < 0.0001*
	HP height (1^st^ M)	0.358	0.229	(0.895–1.798)	*p* < 0.0001*

*HP* Hard palate

*1st PM* Region of the first premolars

*1st M* Region of the first molars

*BC* Beta coefficient

*SE* Standard error

*CI* 95% Confidence interval for beta

^*^Significant association (*p* < 0.05), according to Multiple linear regression

## Discussion

The hard palate is one of the central structures of the stomatognathic system, and it presents great anatomical and clinical importance, as it assists in different orofacial functions, including breathing [1, 5]. When there is an imbalance between the structures of the stomatognathic system due to airway obstruction, or due to changes in the direction of growth and development of the face, the hard palate may change and adapt its morphology, position, and function [2, 3, 5]. In this study, the dimensions of width and height of the posterior region of the hard palate in patients of different sexes, and skeletal and breathing patterns were assessed. It was also investigated if there was an association between the measurements of the hard palate and the volumes of the upper airways and maxillary sinuses. It was found that sex and facial type influence the dimensions of the hard palate, and there was an association between the measurements of the hard palate and the volumes of the maxillary sinuses and upper airways.

About the morphometric analysis of the hard palate, significant differences were found between sexes, and among the facial types, for some measurements. Males had greater width of the hard palate at the first molars region, and greater values of height of the hard palate at the first premolars region, corroborating previous studies [20, 21]. For facial types, in general, dolichocephalic individuals presented greater height of the hard palate in the first premolars and molars regions, whereas brachycephalic patients had greater width of the hard palate in the first premolars region. In view of this result, it is possible to observe the influence of the vertical growth trend in the shape of the hard palate, since the greater vertical tendency of growth (dolichocephalic) presented greater height of the hard palate, while the greater horizontal tendency (brachycephalic) showed greater width of the hard palate. These results corroborate the theories presented by Vucic et al. [2] and Miranda-Viana et al. [3] that the bone structures of the craniofacial complex adapt to variations in craniofacial growth trends. Conversely, two previous studies reported no significant differences among facial types for linear measurements of the hard palate [22, 23]. However, these studies were performed on plaster models, which present deformations inherent to molding and plastering, while in our study the hard palate was evaluated by means of CBCT scans, which provide three-dimensional images with no magnification or distortion. No significant differences were identified among the different skeletal malocclusions for the hard palate morphometry. This may be related to the fact that the skeletal malocclusions are defined by the position of the mandible in relation to the base of the skull, not influencing the width and/or height of the hard palate. To our knowledge, no other study in the literature has performed this type of analysis. Therefore, further studies are encouraged in order to confirm or refute our hypothesis. In regard to the breathing pattern, no significant difference was observed between mouth and nasal breathing for the morphometric analysis of the hard palate, which is in disagreement with prior researches [6, 7, 24]. These different findings are believed to have occurred because these studies were carried out using plaster models. Another factor is that, differently from our sample of adult patients, these studies were carried out in children, in which the bone and integumentary tissues are not yet fully developed. Thus, we believe that the body may adapt to the breathing pattern after the individual is fully grown.

A linear regression was developed to investigate a possible relationship between the hard palate measurements and the volumes of the upper airways and maxillary sinuses. It was found an association between the width and height of the hard palate at the level of the first premolars and the volumes of the upper airways (total volume) and nasopharynx; and between the hard palate width and the volume of the oropharynx. According to our results, the greater the height of the hard palate, the lower the volume of the upper airways; and the greater the width of the hard palate, the higher the volume of the upper airways. It was also found an association between the width and height of the hard palate at the level of the first molars and the volume of the maxillary sinuses, in which the greater the width and height of the hard palate, the greater the volume of the maxillary sinuses. Grauer et al. [25] and Gupta et al. [26] reported an association between the facial width (distance from right to left zygomatic bone) and the upper airway volume. Their results seem to corroborate those of the present study, in which the width of the hard palate showed to be related with the volume of the upper airways, since, although we have measured different bones, both studies assessed the horizontal dimensions of craniofacial structures. Regarding the association between the hard palate and the volume of the maxillary sinuses, this is the first work to perform this analysis. Thus, it is not possible to compare our results with the literature. Therefore, future studies evaluating this association are encouraged, since the results reported here may contribute to additional clinical information, and to assist in the elaboration of the treatment planning in different areas, such as orthodontics, surgery, and otorhinolaryngology.

The authors consider that the relationship between the linear measurements of the hard palate at the first premolars region and the volume of the upper airways may be due to the craniofacial growth trend. Dolichocephalic patients have a tendency of vertical craniofacial growth, presenting a narrower and deeper hard palate. On the other hand, brachycephalic patients have a tendency of horizontal craniofacial growth, presenting a regular and larger hard palate [3, 22, 24]. This is in concordance with the results of this study, in which dolichocephalic and brachycephalic individuals presented greater values of height and width of the hard palate, respectively, at this region. In addition, the study of Fernandes et al. [27] observed a trend towards vertical growth when the oropharynx permeability is reduced. In our research, dolichocephalic patients presented lower volume of the airways at the oropharynx region than brachycephalics. Given these characteristics, there is an indication that the width and height of the posterior region of the hard palate are associated with the volume of the upper airways, as patients with greater height and width of the hard palate presented lower and greater values of volume, respectively.

One may consider that the cross-sectional area of some segments of the upper airways, especially their narrower part, may influence the resistance to the airflow more significantly than their volume. In the present study, we opted to evaluate the volume of the airways instead of measuring cross-sectional areas, since we did not aim at evaluating if there is a relationship between the hard palate dimensions and a possible increased resistance to the airflow. However, one of the findings in this study was the association between the width of the hard palate and the volume of the oropharynx, in which the lower the width of the hard palate, the lower the volume of the oropharynx. Because previous studies in the literature [28, 29] have reported that the oropharynx has the narrower area of the upper airways, it is possible that there is a relationship between the width of the hard palate and the cross-sectional area of the oropharynx as well, i.e., a lower width of the hard palate may be related to an increased resistance to the airflow. However, further studies are necessary to confirm or refute this hypothesis.

In view of the presented and discussed results, it is important to highlight that the sample size of this study was representative of the assessed population, and it was homogeneously distributed within each variable evaluated. Thus, it is considered that individual variations did not affect the study’s results and conclusions. In addition, we understand that the association of the linear dimensions (width and height) of the posterior region of the hard palate with the volumes of the upper airways and maxillary sinuses shows the clinical and anatomical importance of this bone, which may provide clinical information for procedures involving the airways and/or the maxillary sinuses.

Therefore, future studies investigating this clinical relationship in the areas of oral and maxillofacial surgery, orthodontics and otorhinolaryngology are encouraged.

## Conclusion

The width and height of the hard palate are influenced by sex and facial type, but not by skeletal malocclusion or breathing pattern. Also, there is an association between these dimensions and the volumes of the upper airways and maxillary sinuses. Thus, professionals should be aware of possible changes in the upper airways and/or maxillary sinuses volumes when surgical procedures are performed in the maxillary region.

## Data Availability

The datasets used and/or analyzed during the current study are available from the corresponding author on reasonable request.

## Abbreviations

3D: Tridimensional
ANOVA: Analysis of variance
C_1_: First cervical vertebra
C_2_: Second cervical vertebra
C_3_: Third cervical vertebra
CBCT: Cone beam computed tomography
FOV: Field of View
IRB: Institutional review board
LCD: Liquid crystal display
mm: Millimeters
RGn: Retrognathic cephalometric point
ROI: Region of interest
